# Factors associated with viral suppression among HIV-positive Kenyan gay and bisexual men who have sex with men

**DOI:** 10.1080/09540121.2018.1510109

**Published:** 2018-08

**Authors:** Colin P. Kunzweiler, Robert C. Bailey, Supriya D. Mehta, Duncan O. Okall, Eve Obondi, Gaston Djomand, Boaz Otieno Nyunya, Fredrick O. Otieno, Susan M. Graham

**Affiliations:** a Division of Epidemiology and Biostatistics, School of Public Health, University of Illinois at Chicago, Chicago, IL, USA; b Nyanza Reproductive Health Society, Kisumu, Kenya; c Division of Global HIV/AIDS and Tuberculosis, Centers for Disease Control and Prevention, Atlanta, GA, USA; d Division of Global HIV/AIDS, Centers for Disease Control and Prevention, Kisumu, Kenya; e Departments of Medicine, Global Health, and Epidemiology, University of Washington, Seattle, WA, USA

**Keywords:** Gay and bisexual men who have sex with men, GBMSM, MSM, HIV, viral suppression, Kenya

## Abstract

The UNAIDS 90-90-90 target has prioritized achieving high rates of viral suppression. We identified factors associated with viral suppression among HIV-positive gay, bisexual, and other men who have sex with men (GBMSM) in Kisumu, Kenya. HIV-positive participants in the *Anza Mapema* study were offered antiretroviral therapy (ART) regardless of CD4 count. HIV viral load was assessed at baseline and after 6 and 12 months of follow-up. Viral suppression was defined as <1,000 copies/mL. Sociodemographic, sexual behaviors, and psychosocial characteristics were assessed via audio computer-assisted self interview. We used generalized estimating equations to estimate the associations between baseline and time-dependent predictors and viral suppression at 6 and 12 months. Seventy-five HIV-positive men were enrolled in the *Anza Mapema* study, of which 63 had at least one viral load measured during follow-up. Among 52 men with a viral load measure at month 6, 37 (71%) were on ART and virally suppressed. Among 59 men with a viral load measure at month 12, 37 (63%) were on ART and virally suppressed. In the final multivariable model, men who reported receptive or versatile sexual position during anal intercourse with a male partner had reduced odds of viral suppression (aOR = 0.20; 95% CI: 0.08–0.50). Greater levels of coping self-efficacy were associated with increased odds of viral suppression (aOR = 1.10; 95% CI: 1.03–1.16). Despite extensive initiation, retention, and adherence support, the rate of viral suppression in this population did not meet the UNAIDS 90-90-90 target (81% for individuals aware of their HIV status). Pervasive stigma against male-male sex, especially men who practice receptive anal sex, may underlie our findings, which highlight the need for advocacy and stigma reduction efforts. Because coping self-efficacy was a protective factor, efforts to promote resilience in addition to healthy sexual identity development may lead to improved care outcomes among GBMSM in this area.

## Introduction

Gay, bisexual, and other men who have sex with men (GBMSM) are disproportionately affected by the HIV epidemic in sub-Saharan Africa (Geibel, Tun, Tapsoba, & Kellerman, 2010; Sanders et al., 2007; van Griensven & Sanders, 2008). In Kenya, more than 15% of all new HIV infections are attributable to male-male sex (NACC, 2014a, 2014b; NASCOP, 2014). In many countries throughout sub-Saharan Africa stigma, discrimination, and criminalization of same-sex behaviors limit GBMSM’s ability to access HIV treatment and care services. Among 537 MSM recruited from Botswana, Malawi, and Namibia, men enrolled for HIV care and treatment, compared to men who were not, had higher odds of fear of seeking health care services and of ever being denied health care services on the basis of sexuality (Fay et al., 2011). In South Africa, 47 MSM who were interviewed or participated in focus group discussions explained that homophobic verbal harassment from healthcare workers had a negative influence on the appropriate use of health care services and resulted in delays or avoidance of treatment for STI or HIV (Lane, Shade, McIntyre, & Morin, 2008). While these data suggest that GBMSM experience strong barriers to utilization of healthcare and HIV treatment services, expanding access to antiretroviral therapy (ART) represents a priority throughout sub-Saharan Africa (amFAR, 2008; Holland et al., 2015; UNAIDS, 2013).

In 2014, the UNAIDS launched the 90-90-90 targets with the intention of increasing awareness of HIV status, access to ART, and viral suppression among individuals who were diagnosed with HIV (UNAIDS, 2014). High rates of viral suppression are attainable at the community and country level and studies have identified numerous individual, interpersonal, and institutional factors that are associated with viral suppression in general population samples (Bulage et al., 2017; Jobanputra et al., 2015; Musinguzi et al., 2017). However, factors associated with viral suppression among GBMSM in rights-constrained settings such as sub-Saharan Africa are not well understood. We launched a longitudinal cohort study called “*Anza Mapema*” (Kiswahili for “Start Early”) in order to implement a program of HIV prevention and care specifically designed for GBMSM in Kisumu, Kenya. The purpose of this analysis was to identify factors associated with viral suppression among HIV-positive GBMSM who were offered treatment in a GBMSM supportive program and followed for 12 months.

## Methods

### Study population

Participants of the *Anza Mapema* study were recruited between August 2015 and September 2016 through snowballing and peer outreach at hotspots through an existing network of 200 GBMSM that was expanded through GBMSM support groups (Plenty, 2012). Trained peer outreach workers also recruited men from mapped hotspots (e.g., bars, discos, hotels) and through their social networks using a script approved by institutional review boards. All men ≥18 years of age, reporting anal or oral intercourse with another man in the previous six months, not participating in another HIV intervention or vaccine study, and not currently enrolled in an HIV care program were eligible for enrollment. Viral suppression was evaluated among 63 seropositive men who enrolled and were followed up in the *Anza Mapema* study.

### ART initiation

Following a test-and-treat approach, all seropositive men were eligible for immediate ART regardless of CD4 count (MoH & NASCOP, 2016). At baseline, study personnel explored each participant’s willingness to initiate or resume ART, as well as any issues that the participant believed would hinder his ability to attend appointments. ART initiation procedures consisted of two pre-ART sessions. During the first session, the counselor assessed the participant’s understanding of HIV/AIDS and ART and addressed any questions or misconceptions. Once the counselor determined that the participant fully understood the material, the counselor scheduled an appointment one week later. During the second pre-ART session, the counselor reviewed any remaining barriers to initiation and adherence to ART and discussed individualized interventions to overcome any such barriers. After confirming that the participant was ready to start ART, a clinical officer dispensed a two-week supply of ART and reviewed the dosing schedule (and how to deal with missed doses), potential drug interactions, and medication storage with the participant. For men who did not immediately initiate ART, the counselor explored willingness to initiate ART at monthly follow-up visits and attempted to increase the participant’s knowledge of ART and reduce barriers to initiating treatment.

### ART counseling and support

ART refill visits were scheduled after two weeks, then monthly for the first six months, and then every two to three months after that, depending on the participant’s viral response and ART adherence. Between one month and one day prior to each scheduled visit or ART refill men received phone calls, short text messages, and peer reminders to confirm or reschedule the upcoming study visit. If a participant missed their visit or ART refill study personnel initiated retention tracing through phone calls, text messages, and home visits.

During each ART refill visit counselors promoted medication adherence through “Next-Step Counseling” (NSC) (Amico et al., 2012). The NSC model is patient-centered and utilizes motivational interviewing techniques designed to enhance the participant’s motivation to change behaviors that impede or maintain behaviors that promote medication adherence (Amico et al., 2012). NSC materials developed by Graham et al. (2015) for the “*Shikamana*” intervention in Coastal Kenya were condensed and all counselors participated in an abridged training period prior to the start of the study. Training included interactive exercises, role-playing, and sample cases in addition to didactic material (Graham et al., 2015). During all NSC adherence counseling sessions, a general six-step procedure was implemented during which the counselor: (1) introduced the purpose of the session; (2) reviewed the participant’s experiences and progress with ART; (3) explored facilitators of and barriers to medication adherence; (4) identified the next step for medication adherence; (5) identified and agreed on a realistic strategy (or strategies) to accomplish the next step related to the medication adherence; and (6) recorded the plan for upcoming NSC sessions.

### Peer navigator support

In addition to counselor-delivered NSC, participants had the option of receiving adherence support from a trained peer navigator. Prior to the start of the *Anza Mapema* study, peer navigators, called “*Washikaji*” (meaning “those who bond or stick together”), completed training using materials developed for the *Shikamana* intervention of Coastal Kenya (Graham et al., 2015). *Washikaji* were HIV-positive GBMSM with experience taking ART, and agreed to disclose their status in order to provide participants with information regarding HIV and ART, empathetic support, and encouragement (Graham et al., 2015). Participants who were struggling with adherence or were overdue for ART refills were asked if they would accept peer navigation. Those who accepted were assigned a *Washikaji*. Due to turnover (some *Washikaji* pursued opportunities beyond the *Anza Mapema* study), and in some instances sub-optimal performance, additional *Washikaji* were trained in year 2 of the study. Regardless of ART status, all HIV-positive men were encouraged to attend “post-test” support group counseling sessions. Trained HIV-positive peers led weekly discussions regarding the barriers HIV-positive GBMSM face in accessing care and treatment and the value of ART as both treatment and prevention. Study clinicians also led discussions regarding the importance of nutrition while taking ART.

### HIV serostatus and plasma viral load measurements

HIV serostatus was determined through a sequential testing algorithm that followed Kenya National AIDS and STI Control Programme guidelines and included two rapid tests: the Colloidal Gold rapid test kit (KHB Shanghai Kehua Bio-engineering Company, Ltd., China), which was later replaced by the Determine HIV-1/2 Test (Abbott Laboratories, Chicago, IL, USA), and the First Response Rapid HIV test kit (Premier Medical Corporation, Pty., Ltd., India) (NASCOP, 2010). All discordant test results were resolved by DNA PCR testing using the Roche CAP/CTM platform (Roche Molecular Diagnostics, Pleasanton, CA, USA).

HIV viral load was measured by the COBAS AmpliPrep/COBAS TaqMan HIV-1 Test version 2.0 (Roche Molecular Diagnostics, Pleasanton, CA, USA) at baseline and after 6 and 12 months of follow-up. This assay has a valid range of 20–10,000,000 copies/mL; quantities ≤20 copies/mL are below the limit of detection. Per Kenyan Ministry of Health guidelines, we defined viral suppression as <1,000 copies/mL for the primary outcome (NASCOP, 2015). To examine the robustness to varying thresholds of viral suppression, we also used <200 copies/mL (per U.S. Department of Health and Human Services guidelines) to indicate viral suppression (Panel on Antiretroviral Guidelines for Adults and Adolescents, 2016).

### Questionnaire interviews

Sociodemographic characteristics, sexual risk behaviors, and psychosocial factors were assessed via audio computer-assisted self interview (ACASI) at baseline, month 6, and month 12 (NOVA Research Company, Silver Spring, Maryland, USA). All study materials were available in DhoLuo, English, and Kiswahili and research personnel were fluent in all three languages.

Alcohol use was assessed using the Alcohol Use Disorders Identification Test (AUDIT; range: 0–40). Participants who responded “never” to: “How often do you have a drink containing alcohol?” were scored as 0. We dichotomized scores at ≥8, which represents harmful or hazardous alcohol use (Babor, Higgins-Biddle, Saunders, & Monteiro, 2001; Maisto, Carey, Carey, Gordon, & Gleason, 2000; Saunders, Aasland, Babor, De La Fuente, & Grant, 1993). Substance use over the last 12 months was assessed using six binary questions adapted from the Drug Abuse Screening Test (DAST-10; range: 0–6) (Maisto et al., 2000; Skinner, 1982). We dichotomized the score at ≥3 for harmful substance use (Kunzweiler et al., 2017).

Social support was assessed using 11 questions from the Medical Outcomes Study (MOS) Social Support scale (range: 0–100) (Sherbourne & Stewart, 1991). Depressive symptoms were collected using the Personal Health Questionnaire-9 (PHQ-9; range: 0–27) and dichotomized at ≥15, which reflects moderately severe or severe depressive symptoms over the last two weeks (Kroenke, 2010; Kroenke, Spitzer, & Williams, 2001).

Several factors have been identified as components of resilience among GBMSM. In this analysis, we examined coping self-efficacy, or an individual’s confidence in their ability to cope effectively with challenges, threats, or traumatic experiences, as a measure of resilience. We adapted nine questions from the Coping Self-Efficacy (CSE) scale (range: 9–36) (Chesney, Neilands, Chambers, Taylor, & Folkman, 2006; Herrick, Stall, Goldhammer, Egan, & Mayer, 2014). Participants were asked: “When things aren’t going well for you, or when you’re having problems, how confident or certain are you that you can do the following….” Participants then rated the extent to which they believed they could perform behaviors important to adaptive coping (e.g., “Find solutions to your most difficult problems.”). Response options included: cannot do at all; slightly certain can do; moderately certain can do; and very certain can do. The CSE scale was administered at month 6 and month 12 and demonstrated high internal reliability at both time periods (Cronbach’s alpha = 0.85 and 0.93, respectively).

A single dichotomous variable using four binary questions of the United States Agency for International Development Health Policy Initiative MSM Trauma Screening Tool was used to assess experiences of verbal insults, physical abuse, forced sex, or verbal threats (Betron, 2009; Egremy, Betron, & Eckman, 2009). We assessed experiences of physical and sexual abuse during childhood using the Childhood Experience of Care and Abuse (CECA) tool (Bifulco, Bernazzani, Moran, & Jacobs, 2005; Bifulco, Brown, & Harris, 1994). Participants’ experiences of physical and sexual abuse during childhood were collected only at baseline. All other psychosocial characteristics were examined as both baseline and time-dependent variables measured during follow-up visits. Time-dependent ART status (initiated vs. not yet started) was captured at all study visits.

### Statistical analysis

Missing data were not imputed, and a complete case analysis approach was used. We report frequencies and proportions for categorical variables and medians and interquartile ranges for non-normally distributed numeric variables. Generalized estimating equations (GEE) with a logit link, exchangeable correlation matrix, and robust standard errors were used to estimate crude and adjusted odds ratios and 95% confidence intervals for associations between baseline and time-dependent predictors and the repeatedly measured dichotomous outcome. Baseline and time-dependent predictors where *p* ≤ 0.10 in bivariable analyses were included in multivariable model building procedures. Highly correlated variables were removed from the model building procedures. Using the quasi-likelihood under the independence model criterion (QIC), we performed an iterative, manual model selection procedure (Cui & Qian, 2007; Pan, 2001). Study follow-up time (month 12 vs. month 6) was included *a priori*. The model with the smallest QIC was retained as the final multivariable model. Viral suppression status at baseline was added to the final multivariable model in order to determine whether covariates remained significant predictors of viral suppression during follow-up independent of baseline viral suppression status. All *p* values were two-sided. All analyses were completed using Stata/SE 14.0 (Stata Corporation, College Station, Texas, USA).

### Ethical approval

The *Anza Mapema* study was approved by the Maseno University Ethics Review Committee (Kisumu, Kenya), the Institutional Review Board of the University of Illinois at Chicago, and the Human Subjects Division of the University of Washington. This project was reviewed in accordance with CDC human research protections procedures and was determined to be human subjects research, but CDC was not engaged. All participants gave informed consent before taking part in the study.

## Results

**Figure 1.**
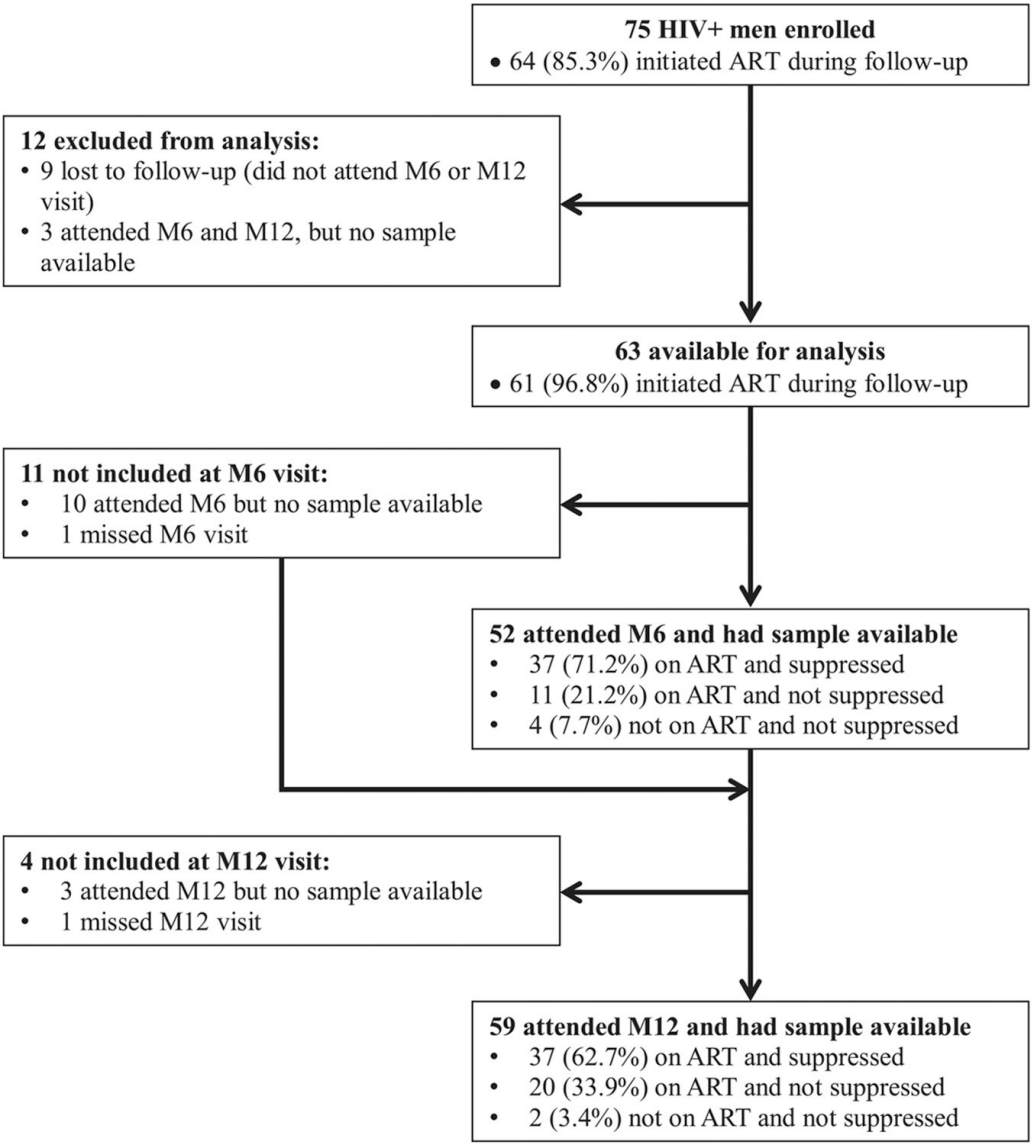
Participant study diagram of HIV-positive men who were enrolled in the *Anza Mapema* study and examined in this analysis. *M6* month 6; *M12* month 12.

Among 711 GBMSM enrolled in the Anza Mapema study, 75 men were HIV-positive at baseline (Figure 1). Among these 75 men, 12 were excluded: 9 were lost to follow-up and did not attend either their month 6 or month 12 visits; 3 attended either their month 6 or month 12 visit, but did not have a biologic sample available to measure viral suppression. The 12 men who were excluded from this analysis did not differ meaningfully by sociodemographic and psychosocial characteristics from the 63 men who had at least one viral load measurement available during follow-up (data not shown). Among these 12 men, four were virally suppressed at baseline and median CD4 was 556 cells/μL (IQR: 438–696); the three men not lost to follow-up all initiated ART prior to month 6 (data not shown).

Table 1 presents the sociodemographic and psychosocial characteristics of the 63 men who had a viral load measurement at one or more follow-up visits and included in this analysis. Median age was 27 years (IQR: 22–32 years), one-third (21 men) had completed ≤8 years of education, and 24 men (38%) were not employed full-time (Table 1). The majority (71%) of men was newly diagnosed and most (80%) had an early stage infection (WHO clinical stage 1) (Table 2). At baseline, median viral load (log 10) was 4.22 (IQR: 1.66–4.69). Nineteen men (31%) were found to be virally suppressed at baseline, of whom 13 reported never having taken ART. Eight men were found to be receiving ART prior to enrollment but without a regular provider, six of whom were virally suppressed. Median CD4 count was 481 cells/μL (IQR: 334–651) and 1 man had a CD4 count <200 cells/μL.

Fifty-two of 63 seropositive men examined in this analysis returned for their month 6 visit and had a viral load result available (Figure 1). Among the 11 men without a viral load measured at month 6, 1 man was receiving ART at baseline, 7 men had initiated ART prior to month 6, and 3 men had not initiated ART. Among the 52 men with a sample available for viral load assessment at month 6, 48 had started ART before their month 6 visit (7 men were receiving ART at baseline; 32 men initiated ART between their month 1 and month 3 visits; 9 men started ART between their month 4 and month 6 visits), while 4 men had not started ART (Table 2). Median viral load (log 10) was 1.54 (IQR: 1.30–3.31) among these 52 men. At month 6, 37 of 52 men (71%) had initiated ART and were suppressed; 11 (21%) had initiated ART but were not sup-pressed; and 4 (8%) had not initiated ART and were not suppressed (Table 2).

Fifty-nine of 63 seropositive men examined in this analysis returned for their month 12 visit and had a viral load result available (Figure 1). The 4 men without a viral load measured at month 12 initiated ART during the study (3 men were receiving ART at baseline and 1 initiated ART prior to month 3). Among the 59 men with a sample available for viral load assessment at month 12, 57 had initiated ART (5 men were receiving ART at baseline; 47 men initiated ART prior to month 6; and 5 men initiated ART prior to month 12), while 2 had not started ART (Table 2). Median viral load (log 10) was 1.49 (IQR: 1.30–3.36) among these 59 men. At month 12, 37 of 59 men (63%) had initiated ART and were suppressed; 20 (34%) had initiated ART but were not suppressed; and 2 (3%) had not initiated ART and were not suppressed (Table 2).

In bivariable analyses, baseline factors associated with reduced odds of viral suppression at *p* ≤ 0.10 included: younger age (18–24 years vs. ≥25 years), less educational attainment (0–8 years vs. ≥13 years), less than full-time employment, transactional sex in the last three months, receptive or versatile (vs. insertive) sexual position during anal intercourse, and unsuppressed viral load at baseline (Table 3). Men who were not living with a male sex partner and men who reported receiving treatment for any STI symptoms in the last three months had increased odds of viral suppression. Regarding time-dependent predictors, men who reported receptive or versatile sexual position during anal intercourse had reduced odds of viral suppression. Men who were not living with a male sexual partner and men who reported greater coping self-efficacy had increased odds of viral suppression (Table 3).

The final multivariable model included both baseline and time-dependent predictors. Men who reported receptive or versatile sexual position during anal intercourse (time-dependent covariate; adjusted odds ratio [aOR] = 0.20; 95% CI: 0.08–0.50) had reduced odds of viral suppression (Table 4). Higher coping self-efficacy scores were associated with increased odds of viral suppression (aOR = 1.10; 95% CI: 1.03–1.16). After controlling for viral suppression status at baseline, usual sexual position during anal intercourse and coping self-efficacy remained significant predictors of viral suppression (Table 4). The results of the secondary outcome (viral suppression defined as <200 copies/mL) were consistent in terms of direction and magnitude with the primary analysis (Table 4).

## Discussion

Despite numerous evidence-based supportive efforts, retention, ART initiation, and viral suppression rates were not optimal among the 75 HIV-positive men enrolled in the *Anza Mapema* study. Overall, 65 (87%) of 75 HIV-positive men returned for their month 6 and month 12 visits, respectively. Sixty-four men (85%) initiated ART during 12 months of follow-up (58 initiated ART before month 6). However, the availability of viral load measurements limited the analysis sample to 63 men (61 initiated ART): 52 at month 6 and 59 at month 12. While 19 men (31%) were already virally suppressed at baseline (31%), only 71% (37/52) were virally suppressed at month 6, and only 63% (37/59) were virally suppressed at month 12. Although the UNAIDS 90-90-90 target for viral suppression is commonly assessed at the community and national level, the proportion of GBMSM examined here who were virally suppressed falls short of this threshold. The rate of viral suppression reported here is also lower than that reported in a systematic review of adult patients in ART programs throughout sub-Saharan Africa after 6 (78%) and 12 months (76%) (Barth, 2010) as well as a recent estimate of viral suppression among adults who had initiated ART in Kenya (75%) (NASCOP, 2014).

The substantial proportion of men who were not virally suppressed but had initiated ART during follow-up, especially the 11 men at month 6, suggests that medication adherence may be a critical barrier to the success of ART in this sample. Sub-optimal ART adherence has been reported in other studies in Kenya and one prior study following HIV-positive adults, including 108 MSM, 15 heterosexual men, and 127 women recruited in Coastal Kenya, reported that a larger proportion of MSM who had started ART reported sub-optimal medication adherence <95% relative to women who had started ART; viral load data were not available (Graham et al., 2013). We did not examine adherence to ART in this analysis for several reasons: (1) viral suppression is the goal of test-and-treat approaches and is a clinically relevant outcome; (2) conceptually, adherence to ART is on the proposed pathway between a test-and-treat approach and viral suppression and including it in modeling procedures could result in faulty measures of association; and (3) examining adherence data among only those who initiated ART would have further reduced the sample available for analysis. In addition to potentially sub-optimal adherence, delays in ART initiation (e.g., 4 men at month 6 and 2 men at month 12 had not initiated ART) also contribute to failure to achieve viral suppression in this sample.

We can only speculate why reported usual sexual position during anal intercourse is associated with viral suppression. Receptive sexual position may be associated with transactional sex, which may influence an individual’s engagement in HIV care and treatment as has been demonstrated in a sample of MSM in Latin America (Biello et al., 2016). Sexual position during anal intercourse may also be related to gender stereotypes and internalized stigma. In Kenya, homophobic sociocultural norms are pervasive and GBMSM are con-tinuously exposed to sociocultural, legal, and structural-level stigma. Men who report insertive sexual position may be less likely to self-identify as gay or homosexual and may be perceived as more masculine or dominant compared to men who report receptive sexual position (Clark et al., 2013; Kippax & Smith, 2001). While we were unable to examine these factors because relevant data were not collected, the role of sexual stigma and internalized homonegativity regarding access to and utilization of HIV care and treatment may be important among men who report receptive or versatile sexual position during anal intercourse.

Resilience has been characterized as a dynamic process by which an individual may positively adapt within contexts of adversity and risk in order to achieve beneficial and avoid negative outcomes (Harper et al., 2015). Individuals may become resilient over time in response to specific challenges or situations by developing both internal and external promotive factors (Chesney et al., 2006; Harper et al., 2015; Herrick et al., 2014). In this analysis, coping self-efficacy, a measure of resilience among GBMSM that reflects one’s confidence in their ability to cope effectively with stressors, was associated with increased odds of viral suppression. Coping self-efficacy, an internal promotive factor of resilience processes, may be especially important in this sample of men who experience intense structural stigma, since men reported limited perceived external social support (median value of MOS Social Support scale = 45; IQR: 32–55) and only a small proportion of men openly discussed their same-sex behaviors with family members or friends (*n* = 9/62; 15%). While coping self-efficacy has not been examined specifically among GBMSM in Kenya, Harper et al. (2015) found that among 511 GBMSM between the ages of 18–29 years in Western Kenya internal promotive factors such as lesbian/gay/bisexual identity and self-esteem were associated with intentions to get tested for HIV and self-reported condom use. These investigators also found that perceived social support, an external promotive factor, was associated with condom use. Due to a high degree of missing data, we were unable to investigate whether HIV-related stigma or failure to disclose HIV status limited the impact of social support in our study population. Factors promotive of resilience processes as they relate to HIV prevention and care should be further examined among GBMSM in sub-Saharan Africa (Millett et al., 2012). Based on the results presented here, interventions that promote coping self-efficacy in response to pervasive homophobia and discrimination may result in improved HIV treatment outcomes such as viral suppression.

The results of this analysis must be interpreted in light of several limitations. First, the small sample size, low rates of visit attendance, and availability of viral load measurements limit statistical power and the measures of association are imprecise. Second, our limited budget did not permit testing for genotypic drug resistance in all participants. In the participants for whom this was done, no drug resistance mutations were identified. However, it is possible that some participants with unsuppressed viral load due to genotypic resistance may have been adherent to treatment. Third, due to the non-probability sampling techniques used during recruitment, men who participated in the study are not representative of GBMSM in Kisumu or Kenya. Fourth, the psychosocial measures used have not been specifically validated among Kenyan GBMSM populations. Fifth, participants may have misreported sexual risk behavior; however, study questionnaires were administered via ACASI, which has been shown to reduce response bias for questions about sensitive behaviors (van der Elst et al., 2009). Finally, some participants likely misrepresented their ART history at baseline, to avoid being excluded from the study.

Rapid linkage to HIV care has been emphasized by test-and-treat approaches to HIV prevention. However, there is limited research that assesses ART initiation and levels of viral suppression over time among GBMSM within rights-constrained settings. The proportion of men who achieved viral suppression in this study falls short of recent estimates of viral suppression among adult patients in ART programs throughout sub-Saharan Africa and the UNAIDS 90-90-90 targets. Stigma and internalized homonegativity among men who report receptive or versatile sexual position during anal intercourse should be further investigated as it relates to viral suppression among GBMSM in Kenya. Given the limited social support reported by the GBMSM examined here, coping self-efficacy represents a crucial promotive factor of resilience for viral suppression. GBMSM in Kenya face substantial sociocultural and legal barriers that adversely impact engagement in HIV care and uptake of ART. While study personnel sought to create an affirming environment and provided numerous services designed to optimize ART initiation, retention, and adherence, overcoming sociocultural and structural barriers in this setting represents a significant challenge to attaining the UNAIDS 90-90-90 targets in this population.

## Figures and Tables

**Table 1.** Distribution of sociodemographic and psychosocial characteristics and sexual risk behaviors among HIV-positive men at baseline (*n* = 63), and by viral suppression status (not suppressed: ≥1,000 copies/mL; suppressed: <1,000 copies/mL) at Month 6 (*n* = 52) and Month 12 (*n* = 59) follow-up visits.

	Baseline	Month 6^a^		Month 12^b^	
Variable	*n* (%)	Not suppressed *n* (%)	Suppressed *n* (%)	Not suppressed *n* (%)	Suppressed *n* (%)
*Sample*	63 (100.0)	15 (28.8)	37 (71.2)	22 (37.3)	37 (62.7)
Age (years) (median/IQR)	27 (22–32)	–	–	–	–
Age (years)					
18–24	24 (38.1)	–	–	–	–
≥25	39 (61.9)	–	–	–	–
Education (years)					
0–8	21 (33.3)	–	–	–	–
9–12	26 (41.3)	–	–	–	–
≥13	16 (25.4)	–	–	–	–
Employment status					
Less than full-time employed	24 (38.1)	6 (40.0)	13 (35.1)	14 (63.6)	14 (37.8)
Full-time employed	39 (61.9)	9 (60.0)	24 (64.9)	8 (36.4)	23 (62.2)
Perceived future financial status					
Uncertain or very uncertain	18 (28.6)	8 (53.3)	13 (35.1)	8 (36.4)	13 (35.1)
Secure or very secure	45 (71.4)	7 (46.7)	24 (64.9)	14 (63.6)	24 (64.9)
Resided in Kisumu for less than 1 year					
No	54 (90.0)	–	–	–	–
Yes	6 (10.0)	–	–	–	–
Marital status					
Single	39 (61.9)	12 (80.0)	23 (63.9)	18 (81.8)	24 (64.9)
Married or living with female partner	9 (14.3)	1 (6.7)	6 (16.7)	1 (4.5)	6 (16.2)
Separated or divorced from female partner	15 (23.8)	2 (13.3)	7 (19.4)	3 (13.6)	7 (18.9)
Currently living with a male sexual partner					
No	38 (60.3)	6 (60.0)	14 (82.4)	13 (59.1)	33 (89.2)
Yes	25 (39.7)	4 (40.0)	3 (17.6)	9 (40.9)	4 (10.8)
Harmful alcohol use (AUDIT ≥8)					
No	29 (46.0)	12 (80.0)	19 (51.4)	15 (68.2)	22 (59.5)
Yes	34 (54.0)	3 (20.0)	18 (48.6)	7 (31.8)	15 (40.5)
Harmful substance use (DAST ≥3)					
No	46 (73.0)	14 (93.3)	31 (83.8)	19 (86.4)	29 (78.4)
Yes	17 (27.0)	1 (6.7)	6 (16.2)	3 (13.6)	8 (21.6)
MOS social support (range: 0–100 scale)	45 (32–55)	59 (34–75)	55 (41–68)	56 (32–75)	57 (50–75)
Coping self-efficacy (range: 9–36)	–	20 (18–25)	26 (21–29)	19 (12–23)	26 (19–32)
Severe depressive symptoms (PHQ-9 ≥ 15)					
No	51 (81.0)	15 (100.0)	35 (94.6)	22 (100.0)	35 (94.6)
Yes	12 (19.0)	0 (0.0)	2 (5.4)	0 (0.0)	2 (5.4)
Any sexual abuse during childhood (CECA)					
No	24 (38.1)	–	–	–	–
Yes	39 (61.9)	–	–	–	–
Experienced physical abuse during childhood (CECA)					
No	24 (38.1)	–	–	–	–
Yes	39 (61.9)	–	–	–	–
Ever refused any services					
No	26 (47.3)	8 (57.1)	25 (73.5)	16 (76.2)	25 (73.5)
Yes	29 (52.7)	6 (42.9)	9 (26.5)	5 (23.8)	9 (26.5)
Experienced recent trauma due to same-sex behaviors (last three months) (USAID HPI)?					
No	16 (31.4)	7 (53.8)	21 (65.6)	17 (81.0)	22 (64.7)
Yes	35 (68.6)	6 (46.2)	11 (34.4)	4 (19.0)	12 (35.3)
Ever had sex with a female partner					
No	17 (27.0)	5 (33.3)	11 (29.7)	11 (50.0)	9 (24.3)
Yes	46 (73.0)	10 (66.7)	26 (70.3)	11 (50.0)	28 (75.7)
Gay or homosexual sexual identity					
No	18 (28.6)	–	–	–	–
Yes	45 (71.4)	–	–	–	–
Transactional sex (participant received money, food, or housing) (last three months)					
No	17 (27.0)	8 (53.3)	18 (48.6)	11 (50.0)	25 (67.6)
Yes	46 (73.0)	7 (46.7)	19 (51.4)	11 (50.0)	12 (32.4)
Always protected AI with a man (last 3 months)					
No	47 (75.8)	6 (40.0)	20 (54.1)	13 (59.1)	19 (51.4)
Yes	15 (24.2)	9 (60.0)	17 (45.9)	9 (40.9)	18 (48.6)
Usual sexual position during AI with a male partner					
Receptive or versatile	33 (54.1)	12 (85.7)	14 (38.9)	16 (72.7)	15 (41.7)
Insertive	28 (45.9)	2 (14.3)	22 (61.1)	6 (27.3)	21 (58.3)
Circumcision status					
No	15 (23.8)	2 (13.3)	10 (27.0)	3 (13.6)	8 (21.6)
Yes	48 (76.2)	13 (86.7)	27 (73.0)	19 (86.4)	29 (78.4)

a Of the 63 men included in this longitudinal analysis, 52 attended their month 6 visit and had a viral load measurement available; 10 attended their month 6 visit but did not have a viral load measurement available; and 1 missed his month 6 visit.

b Of the 63 men included in this longitudinal analysis, 59 attended their month 12 visit and had a viral load measurement available; 3 attended their month 12 visit but did not have a viral load measurement available; and 1 missed his month 12 visit.

*IQR* interquartile range; *AUDIT* Alcohol Use Disorders Identification Test; *DAST* Drug Abuse Screening Test; *MOS* Medical Outcomes Study; *PHQ-9* Personal Health Questionnaire-9; *CECA* Childhood Exposure to Care and Abuse; *USAID HPI* United States Agency for International Development Health Policy Initiative; *AI* anal intercourse.

**Table 2.** Distribution of clinical characteristics among HIV-positive men at baseline (*n* = 63), and by viral suppression status (not suppressed: ≥1,000 copies/mL; suppressed: <1,000 copies/mL) at Month 6 (*n* = 52) and Month 12 (*n* = 59) follow-up visits.

	Baseline	Month 6^a^		Month 12^b^	
Variable	*n* (%)	Not suppressed *n* (%)	Suppressed *n* (%)	Not suppressed *n* (%)	Suppressed *n* (%)
*Sample*	63 (100.0)	15 (28.8)	37 (71.2)	22 (37.3)	37 (62.7)
Received treatment for any STI symptoms (last 3 months)					
No	57 (90.5)	13 (86.7)	33 (89.2)	20 (90.9)	33 (89.2)
Yes	6 (9.5)	2 (13.3)	4 (10.8)	2 (9.1)	4 (10.8)
Any CT and/or NG infection?					
No	45 (72.6)	15 (100.0)	36 (97.3)	17 (81.0)	29 (90.6)
Yes	17 (27.4)	0 (0.0)	1 (2.7)	4 (19.0)	3 (9.4)
HIV diagnosis status					
Previously diagnosed and out-of-care	18 (28.6)	–	–	–	–
Newly diagnosed and out-of-care	45 (71.4)	–	–	–	–
WHO clinical stage					
WHO clinical stage 1	49 (80.3)	14 (100.0)	29 (78.4)	19 (86.4)	30 (81.1)
WHO clinical stage 2, 3, or 4	12 (19.7)	0 (0.0)	8 (21.6)	3 (13.6)	7 (18.9)
CD4 count (median/IQR)	481 (334–651)	517 (306–685)	625 (453–782)	400 (337–733)	581 (433–793)
CD4 count					
≥200	59 (98.3)	14 (93.3)	33 (97.1)	21 (95.5)	35 (100.0)
<200	1 (1.7)	1 (6.7)	1 (2.9)	1 (4.5)	0 (0.0)
Viral load (copies/mL) (median/IQR)	16,798 (46–48,934)	29,456 (6,294–81,396)	20 (20–52)	21,848 (4,260–53,324)	20 (20–20)
Viral load (log10) (median/IQR)	4.22 (1.66–4.69)	4.47 (3.80–4.91)	1.30 (1.30–1.72)	4.33 (3.63–4.73)	1.30 (1.30–1.30)
Viral suppression at baseline					
Not suppressed (≥1,000 copies/mL)	43 (69.4)	–	–	–	–
Suppressed (<1,000 copies/mL)	19 (30.6)	–	–	–	–
ART status					
No	55 (87.3)	4 (26.7)	0 (0.0)	2 (9.1)	0 (0.0)
Yes	8 (12.7)	11 (73.3)	37 (100.0)	20 (90.9)	37 (100.0)

a Of the 63 men included in this longitudinal analysis, 52 attended their month 6 visit and had a viral load measurement available; 10 attended their month 6 visit but did not have a viral load measurement available; and 1 missed his month 6 visit.

b Of the 63 men included in this longitudinal analysis, 59 attended their month 12 visit and had a viral load measurement available; 4 attended their month 12 visit but did not have a viral load measurement available; and 1 missed his month 12 visit.

*STI* sexually transmitted infection; *CT Chlamydia trachomatis; NG Neisseria gonorrhea; WHO* World Health Organization; *IQR* interquartile range; *ART* antiretroviral therapy.

**Table 3.** Unadjusted analysis of baseline and time-dependent factors associated with viral suppression.

Variable	Baseline factors		Time-dependent factors	
	OR (95% CI)	*p* value	OR (95% CI)	*p* value
Age (years)
18–24	0.28 (0.10–0.80)	0.02	–	–
≥25	1.00 (ref)		–	
Education (years)				
0–8	0.20 (0.05–0.82)	0.03	–	–
9–12	0.36 (0.09–1.36)	0.13	–	–
13 or more	1.00 (ref)		–	
Employment				
Less than full-time employed	0.29 (0.10–0.84)	0.02	0.58 (0.29–1.18)	0.13
Full-time employed	1.00 (ref)		1.00 (ref)	
Perceived future financial status				
Uncertain or very uncertain	1.12 (0.39–3.23)	0.84	1.11 (0.56–2.21)	0.76
Secure or very secure	1.00 (ref)		1.00 (ref)	
Resided in Kisumu for less than 1 year				
Yes	1.17 (0.19–7.02)	0.87	–	–
No	1.00 (ref)		–	
Marital status				
Married or living with female partner	1.93 (0.35–10.57)	0.45	2.16 (0.92–5.11)	0.08
Separated or divorced from female partner	0.84 (0.25–2.83)	0.78	1.62 (0.66–3.97)	0.29
Single	1.00 (ref)		1.00 (ref)	
Currently living with a male sexual partner				
No	3.25 (1.15–9.19)	0.03	1.39 (1.00–1.91)	0.05
Yes	1.00 (ref)		1.00 (ref)	
Harmful alcohol use (AUDIT ≥ 8)				
No	1.33 (0.48–3.66)	0.58	0.66 (0.40–1.10)	0.11
Yes	1.00 (ref)		1.00 (ref)	
Harmful substance use (DAST ≥ 3)				
Yes	1.30 (0.39–4.30)	0.67	0.67 (0.23–1.94)	0.46
No	1.00 (ref)		1.00 (ref)	
MOS social support (continuous)	1.02 (0.99–1.05)	0.12	1.00 (0.99–1.01)	0.60
Coping self-efficacy (continuous)	–	–	1.08 (1.03–1.13)	<0.01
Severe depressive symptoms (PHQ-9 ≥ 15)				
Yes	1.35 (0.36–4.99)	0.66	*	*
No	1.00 (ref)		*	
Any sexual abuse during childhood (CECA)				
Yes	1.32 (0.47–3.67)	0.60	–	–
No	1.00 (ref)		–	
Any physical abuse during childhood (CECA)				
Yes	1.10 (0.40–3.05)	0.85	–	–
No	1.00 (ref)		–	
Ever refused any services				
Yes	0.73 (0.25–2.14)	0.57	0.95 (0.59–1.55)	0.85
No	1.00 (ref)		1.00 (ref)	
Experienced recent trauma due to same-sex behaviors (last three months) (USAID HPI)				
Yes	0.93 (0.30–2.94)	0.91	0.99 (0.48–2.05)	0.99
No	1.00 (ref)		1.00 (ref)	
Ever had sex with a female partner				
Yes	1.57 (0.53–4.66)	0.42	1.50 (0.83–2.72)	0.18
No	1.00 (ref)		1.00 (ref)	
Gay or homosexual sexual identity				
No	1.23 (0.39–3.83)	0.72	–	–
Yes	1.00 (ref)		–	
Transactional sex (for which the participant received money, food, or housing) (last 3 months)				
Yes	0.30 (0.09–1.06)	0.06	0.83 (0.53–1.31)	0.43
No	1.00 (ref)		1.00 (ref)	
Always protected AI with a man (last 3 months)				
No	1.36 (0.44–4.20)	0.60	1.26 (0.65–2.46)	0.49
Yes	1.00 (ref)		1.00 (ref)	
Usual sexual position during AI with a male partner				
Receptive or versatile	0.25 (0.08–0.78)	0.02	0.35 (0.17–0.71)	<0.01
Insertive	1.00 (ref)		1.00 (ref)	
Circumcision status				
No	1.91 (0.53–6.85)	0.32	2.12 (0.66–6.86)	0.21
Yes	1.00 (ref)		1.00 (ref)	
Received treatment for any STI symptoms (last 3 months)				
Yes	6.17 (0.81–46.99)	0.08	0.50 (0.19–1.31)	0.16
No	1.00 (ref)		1.00 (ref)	
Any CT and/or NG infection				
Yes	1.84 (0.56–6.06)	0.31	0.32 (0.07–1.56)	0.16
No	1.00 (ref)		1.00 (ref)	
HIV diagnosis status				
Newly diagnosed and out-of-care	0.37 (0.11–1.31)	0.12	–	–
Previously diagnosed and out-of-care	1.00 (ref)		–	
WHO clinical stage				
WHO clinical stage 2, 3, or 4	2.90 (0.72–11.74)	0.14	–	–
WHO clinical stage 1	1.00 (ref)		–	
CD4 count (cells/μL) (continuous)	1.00 (1.00–1.00)	0.26	–	–
CD4 count (cells/μL)				
≥200	*	*	–	–
<200	*		–	
Viral load (log10) at baseline (continuous)	0.56 (0.37–0.85)	0.01	–	–
Viral suppression at baseline				
Not suppressed (≥1,000 copies/mL)	0.11 (0.02–0.58)	0.01	–	–
Suppressed (<1,000 copies/mL)	1.00 (ref)		–	
Time				
Month 12	–	–	0.84 (0.56–1.27)	0.41
Month 6	–		1.00 (ref)	

* Bivariable model was not estimable.

*OR* odds ratio; *CI* confidence interval; *IQR* interquartile range; *AUDIT* Alcohol Use Disorders Identification Test; *DAST* Drug Abuse Screening Test; *MOS* Medical Outcome Study; *PHQ-9* Personal Health Questionnaire-9; *CECA* Childhood Exposure to Care and Abuse; *USAID HPI* United States Agency for International Development Health Policy Initiative; *AI* anal intercourse; *STI* sexually transmitted infection; *CT Chlamydia trachomatis; NG Neisseria gonorrhea; WHO* World Health Organization.

**Table 4.** Adjusted^a^ analysis of factors associated with viral suppression.

	Primary suppression outcome: <1,000 copies/mL vs. ≥1,000 copies/mL				Secondary suppression outcome: <200 copies/mL vs. ≥200 copies/mL			
	Final model^b^		Adjusted for baseline viral suppression^c^		Final model^b^		Adjusted for baseline viral suppression^c^	
Variable	aOR (95% CI)	*p* value	aOR (95% CI)	*p* value	aOR (95% CI)	*p* value	aOR (95% CI)	*p* value
Employment status^d^								
Less than full-time employed	0.39 (0.13–1.16)	0.09	0.41 (0.12–1.46)	0.17	0.32 (0.10–0.95)	0.04	0.31 (0.09–1.09)	0.07
Full-time employed	1.00 (ref)		1.00 (ref)		1.00 (ref)		1.00 (ref)	
Received treatment for any STI symptoms (last 3 months)^d^								
No	0.13 (0.01–1.57)	0.11	0.20 (0.02–2.31)	0.20	0.12 (0.01–1.62)	0.11	0.17 (0.02–2.02)	0.16
Yes	1.00 (ref)		1.00 (ref)		1.00 (ref)		1.00 (ref)	
Usual sexual position during AI with a male partner^e^								
Receptive or versatile	0.20 (0.08–0.50)	<0.01	0.15 (0.05–0.40)	<0.01	0.22 (0.10–0.52)	<0.01	0.19 (0.08–0.48)	<0.01
Insertive	1.00 (ref)		1.00 (ref)		1.00 (ref)		1.00 (ref)	
Coping self-efficacy (continuous)^e^	1.10 (1.03–1.16)	<0.01	1.11 (1.03–1.19)	<0.01	1.09 (1.02–1.16)	0.01	1.10 (1.03–1.17)	0.01
Time								
Month 12	0.71 (0.39–1.30)	0.27	0.70 (0.35–1.41)	0.32	0.83 (0.49–1.40)	0.48	0.85 (0.48–1.53)	0.59
Month 6	1.00 (ref)		1.00 (ref)		1.00 (ref)		1.00 (ref)	
Viral suppression at baseline								
Not suppressed (≥1,000 copies/mL)	–	–	0.27 (0.05–1.35)	0.11	–	–	0.16 (0.02–1.04)	0.06
Suppressed (<1,000 copies/mL)	–		1.00 (ref)		–		1.00 (ref)	

a Predictors are mutually adjusted for all other variables.

b Final multivariable model sample: 63 individuals contributed 108 observation points across two follow-up visits.

c Final multivariable model sample: 62 individuals contributed 106 observation points across two follow-up visits; one individual missing viral load at baseline.

d Time-independent variable collected at baseline.

e Time-dependent variable collected at two follow-up visits.

*aOR* adjusted odds ratio; *CI* confidence interval; *STI* Sexually transmitted infection; *AI* anal intercourse.

## Data Availability

Researchers requesting access to data/resources will be asked to submit a request in writing describing their qualifications including their certification by their local IRB, analytic plans and other uses of the data/resources, and plans to secure the confidentiality and safety of the data. They will be required to agree in writing that they will not share the data with others, will use it only for the research purpose(s) delineated and will return or destroy the data on completion. In order to maintain protection of our participants’ privacy, no directly identifying information will be shared with outside investigators. Given the sensitive nature of the data we are collecting, including HIV diagnosis and same-sex behaviors, no public access file is available.
